# Investigating the role of Rts1 in DNA replication initiation

**DOI:** 10.12688/wellcomeopenres.13884.1

**Published:** 2018-03-06

**Authors:** Ana B.A. Wallis, Conrad A. Nieduszynski

**Affiliations:** 1 Sir William Dunn School of Pathology, University of Oxford, Oxford, Oxfordshire, OX1 3RE, UK

**Keywords:** DNA Replication, Phosphatases, Rts1, Rif1, Orc2

## Abstract

**Background:** Understanding DNA replication initiation is essential to understand the mis-regulation of replication seen in cancer and other human disorders. DNA replication initiates from DNA replication origins. In eukaryotes, replication is dependent on cell cycle kinases which function during S phase. Dbf4-dependent kinase (DDK) and cyclin-dependent kinase (CDK) act to phosphorylate the DNA helicase (composed of mini chromosome maintenance proteins: Mcm2-7) and firing factors to activate replication origins. It has recently been found that Rif1 can oppose DDK phosphorylation. Rif1 can recruit protein phosphatase 1 (PP1) to dephosphorylate MCM and restricts origin firing. In this study, we investigate a potential role for another phosphatase, protein phosphatase 2A (PP2A), in regulating DNA replication initiation. The PP2A regulatory subunit Rts1 was previously identified in a large-scale genomic screen to have a genetic interaction with *ORC2 *(a DNA replication licensing factor). Deletion of *RTS1* synthetically rescued the temperature-sensitive (ts-) phenotype of *ORC2 *mutants.

**Methods:** We deleted *RTS1 *in multiple ts-replication factor *Saccharomyces cerevisiae *strains, including *ORC2*.  Dilution series assays were carried out to compare qualitatively the growth of double mutant *∆rts1* ts-replication factor strains relative to the respective single mutant strains.

**Results:** No synthetic rescue of temperature-sensitivity was observed. Instead we found an additive phenotype, indicating gene products function in separate biological processes. These findings are in agreement with a recent genomic screen which found that *RTS1* deletion in several ts-replication factor strains led to increased temperature-sensitivity.

**Conclusions:** We find no evidence that Rts1 is involved in the dephosphorylation of DNA replication initiation factors.

## Introduction

Errors during DNA replication can lead to aneuploidy and DNA damage ( Passerini *et al.*, 2016). An insufficient concentration of replication factors can also lead to genomic instability ( Orr *et al.*, 2010). Therefore, it is important that cells ensure that a single round of DNA replication occurs in each cell cycle. DNA replication initiates from DNA replication origins (origins). In *Saccharomyces cerevisiae* origins are formed of an autonomously replicating sequence (ARS) which contains an 11bp ARS consensus sequence (ACS). Origins recruit the origin recognition complex (ORC) via the ACS, which in turn facilitates origin licensing. Origin licensing factors (Cdc6 and Cdt1) bind at the origin and allow the mini-chromosome maintenance (MCM) proteins to also bind. Post-licensing, firing factors (Cdc45, Sld2, Sld3, Dpb11) recruit the loading complex which contains GINS (a four-subunit complex), Cdc45 and the replicative polymerases (Polε, Polδ and Polα) ( Yeeles *et al.*, 2015). Cdc45, MCM and GINS collectively form the CMG ( Yeeles *et al.*, 2015). The CMG melts DNA, unwinding the DNA double helix to allow loading of the polymerases, to begin DNA replication. To prevent re-replication, origin licensing in eukaryotes is limited to G1 phase of the cell cycle, and origin firing is restricted to S phase ( Blow & Dutta, 2005). In *S. cerevisiae*, loss of DNA re-replication control leads to genome instability including gene amplification ( Green *et al.*, 2010).

The activities of licensing and firing factors are influenced by cell cycle kinases. For example, origin firing is dependent upon two kinases: the Dbf4-dependent kinase (DDK) and the cyclin-dependent kinase (CDK). DDK phosphorylates multiple chromatin-bound MCM subunits, including Mcm4 and Mcm6. Phosphorylation facilitates Sld3, Sld7 and Cdc45 binding. Subsequently, CDK phosphorylates Sld3 and Sld2, which then recruits the loading complex ( Zegerman, 2015), which leads to origin firing.

However, the kinase-driven view of replication initiation outlined above is now known to be incomplete ( Davé *et al.*, 2014). A role for dephosphorylation in controlling DNA replication initiation was established recently ( Davé *et al.*, 2014; Hiraga *et al.*, 2014; Mattarocci *et al.*, 2014; Poh *et al.*, 2014). The Rap1-interacting factor (Rif1) is able to recruit protein phosphatase 1 (PP1) to MCM subunits and dephosphorylate them ( Davé *et al.*, 2014; Hiraga *et al.*, 2014; Mattarocci *et al.*, 2014; Poh *et al.*, 2014). A greater DDK concentration is therefore required to promote origin firing, since the MCM phosphorylation rate must exceed its dephosphorylation rate. Conversely, DDK can bind directly to Rif1 and inhibit its interaction with PP1 ( Hiraga *et al.*, 2014). Therefore, as DDK levels increase during S phase, MCM phosphorylation is promoted and dephosphorylation is inhibited. The resulting feedback loop allows for a rapid switch from low MCM phosphorylation in G1 to high MCM phosphorylation in S phase.

Rif1-PP1 involvement in DNA replication control appears to be conserved throughout eukaryotes, both *Xenopus* egg extract and HeLa cell studies support the findings in yeast ( Poh *et al.*, 2014; Yamazaki *et al.*, 2012). Additionally, there is evidence that Rif1-PP1 controls further aspects of DNA replication initiation. For example, in yeast, Rif1-PP1 may antagonise CDK phosphorylation ( Stark *et al.*, 2015). In *RIF1* null yeast strains phosphorylation of Sld3, but not Sld2, is increased ( Mattarocci *et al.*, 2014). Deletion of *RIF1* can partially rescue the phenotype of temperature-sensitive (ts-) origin firing factor alleles including Dpb11, Cdc45 and Sld3 ( Mattarocci *et al.*, 2014). In human cells, Rif1-PP1 is active during mitotic exit. Dephosphorylation of Orc2, an ORC subunit, allows the process of origin licensing to start again. Human Rif1-PP1 not only antagonises MCM phosphorylation, but also positively promotes DNA replication origin licensing ( Hiraga *et al.*, 2017).

Before a role for Rif1-PP1 in DNA replication was described, Rif1 was known to be a telomere-associated protein, contributing to the late replication of telomeric regions ( Lian *et al.*, 2011). Rif1 has also been implicated in a PP1-independent role in DNA replication at the whole genome level. The conserved replication timing of some genomic domains is altered in *RIF1* mutant cells due to disordered chromatin organisation. These observations led to a role for Rif1 in physically grouping similarly timed replication domains being described ( Foti *et al.*, 2016).

The importance of Rif1-PP1 dephosphorylation raises the question of whether other phosphatases are implicated in DNA replication control. A large genomic screen for genetic interactions previously identified a potential synthetic rescue of mutant *ORC2* by additional *RTS1* deletion ( Costanzo *et al.*, 2010). Rts1 is a regulatory subunit for the PP2A phosphatase, which has been previously implicated in DNA replication. PP2A antagonises the DNA damage checkpoint protein Chk1 ( Petersen *et al.*, 2006), and its function is required for Cdc45 loading onto chromatin ( Chou *et al.*, 2002; Peplowska *et al.*, 2014). Whether this interaction is direct, occurs via dephosphorylation of Sld3 ( Guo *et al.*, 2015), or uses another protein complex ( Chowdhury *et al.*, 2010) is as yet unclear. It has also been proposed that another regulatory subunit of PP2A, PR48, allows it to bind to and dephosphorylate the licensing factor Cdc6 during mitotic exit, promoting origin licensing during G1 ( Yan *et al.*, 2000). Unlike PP1, which in humans is regulated by more than 90 different subunits, PP2A has only 13 regulatory subunits in humans, and 3 in yeast ( Stark *et al.*, 2015).

Whilst Rif1 is associated with telomeres and late-replicating regions of DNA, Rts1 is associated with the protection of centromeres, which are known to replicate early ( Mccarroll & Fangman, 1988). Rts1-PP2A is enriched at centromeres pre-anaphase promoting cell cycle progression after appropriate microtubule binding, and correct chromosome segregation ( Peplowska *et al.*, 2014).

This study investigates a putative role for Rts1-PP2A, akin to Rif1-PP1, in controlling DNA replication licensing and firing. We use a panel of ts-replication factor mutants to screen for synthetic rescue by *RTS1* deletion. We find that *RTS1* deletion using classical genetics does not alleviate the lethality caused by inactivating origin initiation factors. Whilst the published synthetic rescue given by *rif1Δ* is confirmed in these strains, we find an additive effect for *r*ts *1Δ*. This indicates that two separate pathways are compromised. Although these data contradict the original genetic interactions screen ( Costanzo *et al.*, 2010) they are in accordance with a more recent screen ( Costanzo *et al.*, 2016), suggesting that *RTS1* deletion results in an enhanced (rather than alleviated) phenotype in some replication factor mutants.

## Methods

### Yeast strains and methods

Yeast strains were cultured both in liquid and on solid YPAD media (CCM1010 and CM0510 respectively; Formedium, Hunstanton, UK), and manipulated according to established practices ( Treco & Winston, 2008). Most yeast strains used had a W303 background. However, strains from the *S. cerevisiae* genome deletion project ( Giaever *et al.*, 2002) had an S288c background. All strains used are listed in Table 1.

**Table 1.** List of yeast strains. A list of yeast strains used in this study.

STRAIN	*GENOTYPE*	SOURCE
T7107	*MATa: RAD5, BUD4, leu2, ura3, trp1, ade2, his3*	T. Tanaka lab
45-1	*MATa: leu2-3, 112 ura3-52 ade2-1 lys2-801 cdc45-1*	C. Nieduszynski lab
CNY167	*MATa: ade2-1 his3-11,15 leu2-3,112 trp1-1 ura3-1* *can1-100 Gal+ orc5-1*	C. Nieduszynski lab
AUY080	*MATa: ade2-1 trp1-1 can1-100 leu2-3,112 his3-11,15* *ura3 GAL+ ssd1,d2 RAD5 orc2-1*	C. Nieduszynski lab
K2539	*MATα: cdc9-1 Backcrossed three times to K699/K700*	T. Tanaka lab
dbf4-1	*MATa: ade2-1 his3-11,15 leu2-3,112 trp1-1 ura3-1* *can1-100 ssd1-d2 Gal+ dbf4-1*	Tanaka & Nasmyth, 1998
YKB2	*MATa: leu2-3,112 trp1-1 can1-100 ura3-1 ade2-1* *his3- 11,15, cdc7-4*	Mattarocci *et al*., 2014
YYK32	*MATa: leu2-3,112 trp1-1 can1-100 ura3-1 ade2-1* *his3- 11,15, cdc45-27, bar1Δ::hisG*	Mattarocci *et al*., 2014
YYK14	*MATa: leu2-3,112 trp1-1 can1-100 ura3-1 ade2-1* *his3- 11,15, sld3-4, bar1Δ::hisG*	Mattarocci *et al*., 2014
YNIG63(2)	*MATa: leu2-3,112 trp1-1 can1-100 ura3-1 ade2-1* *his3- 11,15 , dpb11-24, bar1Δ::hisG*	Mattarocci *et al*., 2014
YCH175	*MATα: ho, ade2, trp1, can1, leu2, his3, GAL, psi +* *W303-1; cdc6-1*	Mattarocci *et al*., 2014
YOR014W	*rts1Δ::KanMX S288c*	Giaever *et al*., 2002
YBR275C	*rif1Δ::KanMX S288c*	Giaever *et al*., 2002
YDR007W	*trp1Δ::KanMX S288c*	Giaever *et al*., 2002
ACY001	W303 *MATα rts1Δ::kanMX*	This Study
ACY004	W303 *MATa rts1Δ::kanMX*	This Study
ACY007	W303 *MATα rif1Δ::kanMX*	This Study
ACY010	W303 *MATa rif1Δ::kanMX*	This Study
ACY013	W303 *MATα trp1Δ::kanMX*	This Study
ACY016	W303 *MATa trp1Δ::kanMX*	This Study
ACY036	W303 *Diploid orc2-1 rts1Δ::kanMX*	This Study
ACY113	W303 *MATα orc2-1 rts1Δ::kanMX*	This Study
ACY044	W303 *Diploid orc2-1 rif1Δ::kanMX*	This Study
ACY100	W303 *MATα orc2-1 rif1Δ::kanMX*	This Study
ACY079	W303 *Diploid cdc6-1 rts1Δ::kanMX*	This Study
ACY112	W303 *MATa cdc6-1 rts1Δ::kanMX*	This Study
ACY081	W303 *Diploid cdc6-1 rif1Δ::kanMX*	This Study
ACY148	W303 *MATa cdc6-1 rif1Δ::kanMX*	This Study
ACY035	W303 *Diploid cdc7-4 rts1Δ::kanMX*	This Study
ACY096	W303 *MATa cdc7-4 rts1Δ::kanMX*	This Study
ACY042	W303 *Diploid cdc7-4 rif1Δ::kanMX*	This Study
ACY139	W303 *MATa cdc7-4 rif1Δ::kanMX*	This Study
ACY071	W303 *Diploid dbf4-1 rts1Δ::kanMX*	This Study
ACY106	W303 *MATα dbf4-1 rts1Δ::kanMX*	This Study
ACY073	W303 *Diploid dbf4-1 rif1Δ::kanMX*	This Study
ACY104	W303 *MATα dbf4-1 rif1Δ::kanMX*	This Study
ACY031	W303 *Diploid cdc45-27 rts1Δ::kanMX*	This Study
ACY093	W303 *MATα cdc45-27 rts1Δ::kanMX*	This Study
ACY037	W303 *Diploid cdc45-27 rif1Δ::kanMX*	This Study
ACY142	W303 *MATa cdc45-27 rif1Δ::kanMX*	This Study
ACY019	W303 *Diploid cdc45-1 rts1Δ::kanMX*	This Study
ACY087	W303 *MATa cdc45-1 rts1Δ::kanMX*	This Study
ACY051	W303 *Diploid cdc45-1 rif1Δ::kanMX*	This Study
ACY145	W303 *MATa cdc45-1 rif1Δ::kanMX*	This Study
ACY025	W303 *Diploid cdc9-1 rts1Δ::kanMX*	This Study
ACY067	W303 *MATα cdc9-1 rts1Δ::kanMX*	This Study
ACY050	W303 *Diploid cdc9-1 rif1Δ::kanMX*	This Study
ACY120	W303 *MATα cdc9-1 rif1Δ::kanMX*	This Study
ACY069	W303 *Diploid dpb11-24 rts1Δ::kanMX*	This Study
ACY087	W303 *MATa dpb11-24 rts1Δ::kanMX*	This Study
ACY046	W303 *Diploid dpb11-24 rif1Δ::kanMX*	This Study
ACY123	W303 *MATα dpb11-24 rif1Δ::kanMX*	This Study

In order to delete *S. cerevisiae* genes, the appropriate *KanMX* deletion cassettes from the SGDP were incorporated into a recipient strain by transformation. Deletion was confirmed by PCR spanning the deletion site. Oligonucleotide sequences are listed in Table 2. Ts-initiation factor mutant strains were confirmed by a lack of growth on solid YPAD plates at restrictive temperatures. Ts-initiation factor mutations with respective permissive and restrictive temperatures are listed in Table 3. Double mutant (ts-mutant / gene deletion) strains were confirmed by temperature-sensitivity and G418 resistance (400 µg/ml G418 disulfate salt; A1720-5G, Sigma-Aldrich, St Louis, MO, USA), relative to wild-type sister colonies.

**Table 2.** List of oligonucleotides. A list of oligonucleotides used in this study.

PRIMER 1	PRIMER 2	PRODUCT
AC0003 TTTTCAGTTCTTTGTGTTTTTCCTC	AC0004 TGATCCTTTAGAATGGAGAAGATTG	*rif1Δ::kanMX*
AC0005 TAAACCATCGTCGCCGTAA	AC0006 GGAAGAAGGAAAGCGAAAAGA	*rts1Δ::kanMX*
CA1118 CCATTACGCTCGTCATCAAA	AC0010 AAGAAACAAGAAGTCAACAGAAGG	Confirms 5’ insertion of *rif1Δ::kanMX*
CA1117 GATAATGTCGGGCAATCAGG	AC0009 GCGGTAGCATTTCCATCATAA	Confirms 3’ insertion of *rif1Δ::kanMX*
CA1118 CCATTACGCTCGTCATCAAA	AC0011 GGCATGTCAATACGTCTCGTT	Confirms 5’ insertion of *rts1Δ::kanMX*
CA1117 GATAATGTCGGGCAATCAGG	AC0012 GGCAAGGTTTACGGAAAAGA	Confirms 3’ insertion of *rts1Δ::kanMX*

**Table 3.** Temperature-sensitive mutation strains used in this study. Mutant forms of replication initiation factors, temperatures at which we observed phenotypes, and the study that originally reported each strain.

TS-REPLICATION FACTOR MUTATION	TEMPERATURE AT WHICH PHENOTYPE OBSERVED	TEMPERATURE AT WHICH NO PHENOTYPE OBSERVED	REFERENCE
*orc2-1*	30	23	Foss *et al*., 1993
*cdc6-1*	33	30	Hartwell *et al*., 1973
*cdc7-4*	30	23	Hartwell *et al*., 1973
*dbf4-1*	32	30	Johnston & Thomas, 1982
*cdc45-27*	32	30	Kamimura *et al*., 2001
*cdc45-1*	23	30	Moir *et al*., 1982
*cdc9-1*	30	23	Hartwell *et al*., 1973
*dpb11-24*	37	32	Tanaka *et al*., 2007

### Dilution series assay

Strains were inoculated into YPAD liquid medium and cultured for 12–16 hours. Haploid cell concentration was inferred from attenuance measured at 600 nm (using a BioMate3 spectrophotometer; Thermofisher, Waltham, MA, USA). Cells were diluted initially to 10 ^7^cells/ml, before serial 10-fold dilutions were prepared. Dilution spots of 5 µl were pipetted onto YPAD agar plates and incubated at stated temperatures. Control strains were included on each plate. After two days, plates were photographed and images were analysed qualitatively by observation of relative growth of yeast strains.

## Results

### *RTS1* deletion does not synthetically rescue *orc2-1*

**Figure 1.**
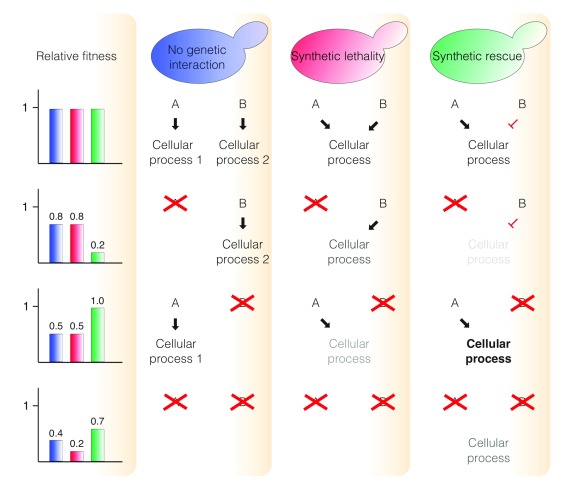
A summary of genetic interactions. Two genes, *A* and *B*, show genetic interactions as a result of the interacting functions of their products: A and B. When A and B function in different cellular processes, the relative fitness of an *A ^-^B ^-^* double mutant is a product of the relative fitness of the two single mutants (no genetic interaction). If the double mutant strain has a lower than expected viability, it is described as synthetic lethality, indicating redundant functions for the two gene products in one cellular process. In contrast, a greater than expected viability (synthetic rescue) indicates that the gene products have opposing roles in a cellular process.

**Figure 2.**
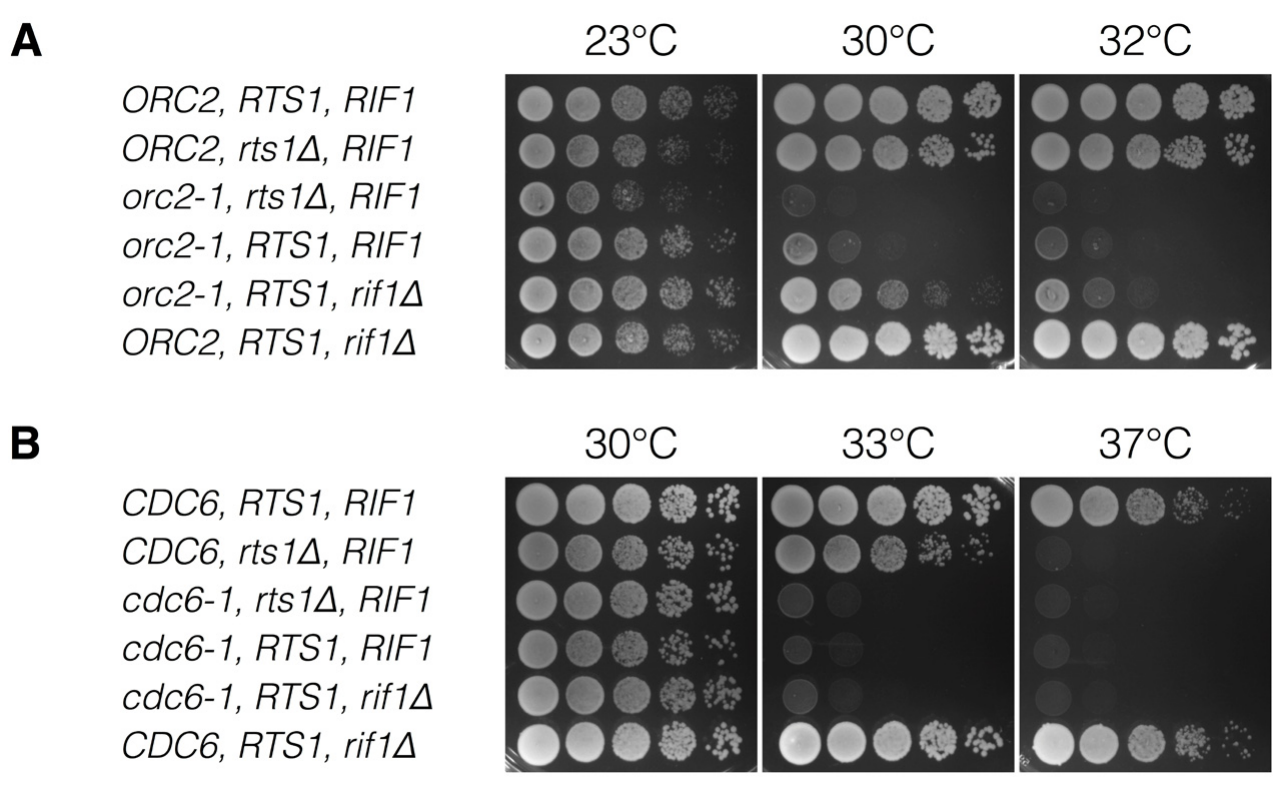
*RTS1* deletion does not suppress temperature-sensitivity of DNA replication origin licensing factor mutants. Budding yeast strains with ts-mutants of replication factors, together with either wild type, *rts1Δ* or *rif1Δ* were characterised by dilution viability assays. Wild type strains, without ts-replication factors, are shown at the top of each panel, as a control. ( **A**) ORC subunit ( *orc2-1*) is assayed. ( **B**) A second pre-RC component, *cdc6-1* is assayed.

The combination of two mutations, which individually decrease cell fitness, can restore fitness if the genes have opposing effects (Synthetic Rescue, Figure 1). In the presence of a replication factor mutant, such as *orc2-1*, even at the permissive temperature origin firing can be reduced by as much as 30% ( Shimada *et al.*, 2002), leading to growth deficiency. We first examined *RTS1* deletion in an *orc2-1* strain ( Figure 2A), since a synthetic rescue phenotype has been reported ( Costanzo *et al.*, 2010). A dilution series viability assay was used to assess synthetic rescue. We found that an *orc2-1 rts1Δ* strain had a more severe ts-phenotype that either the *orc2-1* or *rts1Δ* strains ( Figure 2A). This additive effect indicates that the two genes are not acting within the same pathway. Conversely, a small synthetic rescue was observed in the *orc2-1 rif1Δ* strain ( Figure 2A). It has previously been shown that *RIF1* deletion leads to slight synthetic rescue in *orc5-1* strains, consistent with this result ( Mattarocci *et al.*, 2014).

### The origin licensing factor Cdc6 is not opposed by *RTS1*

Since Rif1 recruits PP1 to oppose DDK phosphorylation, *RIF1* deletion provides limited or no rescue to the temperature sensitivity of pre-Replication Complex (pre-RC) factors mutants, which function prior to DDK ( Mattarocci *et al.*, 2014). Given that this study aimed to investigate a role for Rts1 in opposing the action of Orc2, we next looked for a genetic interaction between *RTS1* and another origin licensing factor: *CDC6*, which loads MCM. Deleting *RTS1* in the context of *cdc6-*1 showed no rescue relative to the original ts-strain ( Figure 2B). Similarly, deletion of *RIF1* gave no synthetic rescue, consistent with published data ( Mattarocci *et al.*, 2014). It has previously been shown that *rts1Δ* yeast strains are ts at 37°C ( Auesukaree *et al.*, 2009; Shu & Hallberg, 1995), while *rif1Δ* strains are not ( Mattarocci *et al.*, 2014). Our study confirmed both these phenotypes ( Figure 2B).

### Rts1 does not antagonise DDK phosphorylation

**Figure 3.**
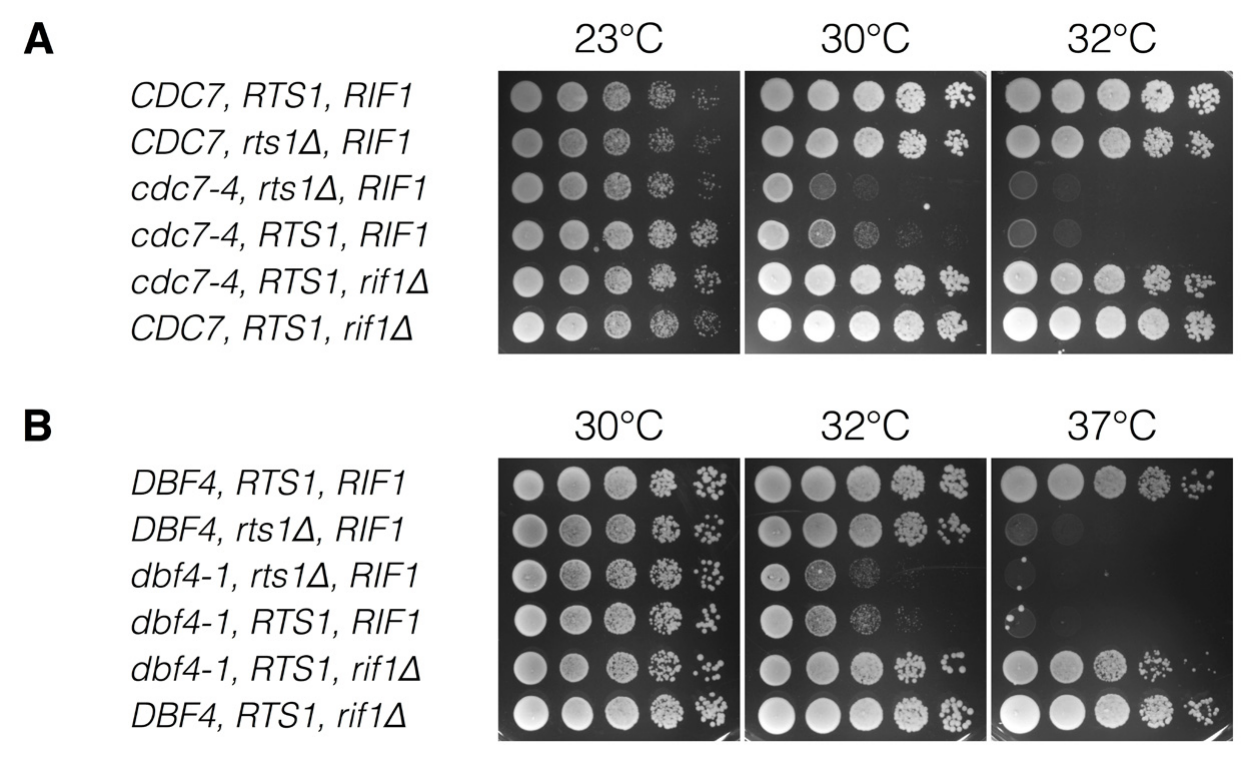
Unlike *rif1Δ*, *rts1Δ* cannot synthetically rescue ts-forms of DDK subunits. Budding yeast strains with ts-mutants of replication factors, together with either wild type, *rts1Δ* or *rif1Δ* were characterised by dilution viability assays. Wild type strains, without ts-replication factors, are shown at the top of each panel, as a control. Ts-forms of DDK subunits, Cdc7 ( **A**) and Dbf4 ( **B**) are assayed.

A potential role for Rts1-PP2A phosphatase in DNA replication initiation could be to oppose the action of a kinase. The established role for Rif1-PP1 in opposing DDK activity indicates that regulation of phosphorylation is key during this step of replication initiation. Therefore, *RTS1* was deleted in combination with ts-forms of both subunits of DDK (Cdc7 and Dbf4). However, *rts1Δ* had a slightly additive effect on temperature-sensitivity in both *cdc7-4* ( Figure 3A) and *dbf4-1* ( Figure 3B) strains. This was in stark contrast to the strong restoration of cell growth at elevated temperatures in *cdc7-4 rif1Δ* ( Figure 3A) and *dbf4-1 rif1Δ* ( Figure 3B) strains. This suggests that Rif1 and Rts1 do not have analogous roles in control of DNA replication initiation, and that Rts1-PP2A does not antagonise DDK activity.

### Replication firing factors are not opposed by Rts1

**Figure 4.**
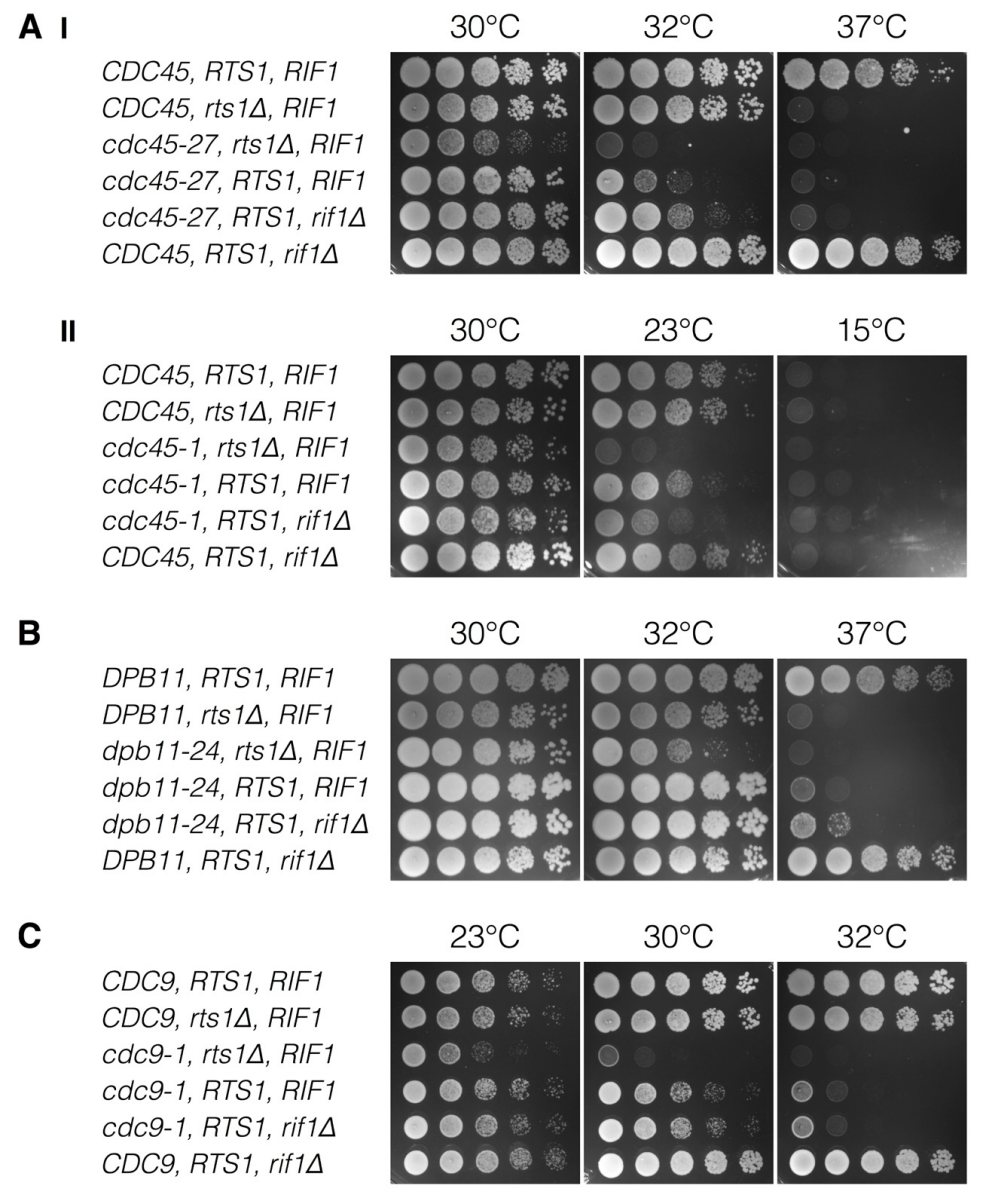
*RTS1* causes synthetic lethality with DNA replication firing factors and a DNA replication progression factor. Budding yeast strains with ts-mutants of replication factors, together with either wild type, *rts1Δ* or *rif1Δ* were characterised by dilution viability assays. Wild type strains, without ts-replication factors, are shown at the top of each panel, as a control. ( **A**) Ts- (I) and cold- sensitive (II) forms of Cdc45, ( **B**) the replication firing factor Dbp11, which functions post-DDK, and ( **C**) Cdc9 ligase, are assayed.

MCM phosphorylation by DDK recruits the firing factor, Cdc45. The sequential recruitment of further firing factors (e.g. Dpb11) relies on phosphorylation by CDK. Therefore, Rts1-PP2A activity could be important following DDK activity, to limit CDK-induced origin firing. In order to test this hypothesis, *RTS1* was deleted in the context of ts- *cdc45-1* and *dpb11-24* firing factors. Unlike *rif1Δ*, *rts1Δ* did not rescue *cdc45-27* or *dpb11-24* temperature-sensitivity ( Figure 4AI, 4B). Conversely, *rts1Δ* led to an increased lethality in these strains, suggesting either an additive or synthetic lethality effect ( Figure 1).

However, the cold-sensitive *cdc45-1* strain (restricted at 15°C) had no phenotypic rescue by either *rif1Δ* or *rts1Δ* ( Figure 4AII). Instead, an additive effect was observed for both gene deletions, which was stronger in the case of *RTS1*. This additive effect of *rif1Δ* in cold-sensitive *cdc45-1* contradicts the known rescue of ts- *cdc45-27* by *rif1Δ* ( Mattarocci *et al.*, 2014), a finding repeated in this study. Therefore, the significance of an additive effect of *rts1Δ* with *cdc45-1* is unclear.

### Synthetic lethality of *RTS1* and *CDC9* ligase

Since the detrimental effect of *RTS1* deletion alongside ts-DNA replication initiation factors appeared to be most severe in factors which function later in the firing process, we hypothesised that Rts1 could play a role in DNA replication post-firing. Therefore, *RTS1* was deleted alongside a ts- *CDC9* ligase allele ( *cdc9-1*). Cdc9 ligates lagging strand Okazaki fragments, aiding in DNA replication during elongation. *RTS1* deletion in a *cdc9-1* strain led to the greatest synthetic lethality, relative to *RTS1* deletion in the other ts-strains examined. No effect of *rif1Δ* was seen in *cdc9-1* cells. This was anticipated, since Rif1 is known to function during replication initiation. These data show that, if Rts1 plays a role in DNA replication, it is not akin to that played by Rif1, and does not appear to oppose critical events leading to origin licensing or origin firing.

## Discussion

In this study, *RTS1*, which encodes a regulatory subunit for the PP2A phosphatase, was deleted in the context of a range of ts-replication factor mutants. At no stage of DNA replication initiation (licensing, DDK-phosphorylation, and origin firing) did deletion of *RTS1* lead to synthetic rescue of ts-phenotypes. In contrast, deletion of *RIF1* was able to rescue ts-mutants of replication firing factors, and some replication licensing factors. Rif1 recruits PP1 phosphatase to DNA replication origins where it counteracts MCM phosphorylation by DDK. When replication origin firing is limited by mutant replication factors, removing this negative regulation allows more replication origins to fire, giving synthetic rescue. In contrast, *rts1Δ* leads to increased temperature-sensitivity when combined with *orc2-1*, *cdc7-4*, *cdc45-1* and *dpb11-24* mutant replication factors. The lack of synthetic rescue given by *rts1Δ* in these strains indicates that there is no evidence for a role for Rts1 in limiting origin firing, analogous to that played by Rif1.

Without a genetic interaction, combining mutations in genes involved in separate pathways will give an additive effect. However, alone, *rts1Δ* has no growth inhibition at temperatures below 34°C. Therefore, the extent of reduced cell viability seen in some double mutant strains, such as *dpb11-24 rts1Δ* at ** 32°C ( Figure 4), suggests synthetic lethality. This may be the result of non-specific protein instability after heat stress, in *rts1Δ* strains. Over-expression of *RTS1* can partially rescue lethality of a ts- *HPS60* allele ( Shu & Hallberg, 1995). Hps60 is a mitochondrial protein that aids in protein refolding after heat stress ( Shu & Hallberg, 1995). A reduced capability to maintain protein structure in heat stress conditions could explain the increased temperature sensitivity of unstable replication factor forms in *rts1Δ* cells. This could be analogous to the partial rescue of the ts-phenotype of *orc2-1* by mutations in the ubiquitin ligase *UBA1* ( Shimada *et al.*, 2002). Therefore, investigation of heat stress in *rts1Δ* strains would be needed to elucidate the molecular mechanism.

The extent of the additional lethality given by *rts1Δ* alongside mutant ts-replication factors is inconsistent. If added lethality of *rts1Δ* depends on the function of the ts-factor, this could indicate a functional genetic interaction between that replication factor and *RTS1*. In DDK and pre-RC factor mutants ( *cdc7-4, dbf4-1, cdc6-1*) there is either mildly increased temperature-sensitivity or no effect given by *rts1Δ* ( Figure 2B, Figure 3). However, in post-DDK acting firing factors *cdc45-1* and *dpb11-24*, the observed increase in the ts-phenotype is larger ( Figure 4). We cannot exclude the possibility that this effect is due to greater heat-instability of the *cdc45-1* and *dpb11-24* mutant replication factors. However, these data may suggest a role for Rts1 late in DNA replication initiation, demonstrating a genuine negative genetic interaction between *RTS1* and post-DDK replication firing factors.

Interestingly, the greatest synthetic lethality is seen between *RTS1* and a replication elongation factor: *CDC9*. Cdc9 is important for DNA replication progression and elongation rather than initiation. Accordingly, we find no synthetic rescue by *rif1Δ* in the *cdc9-1* strain ( Figure 4). The observed synthetic lethality of *RTS1* deletion in a *cdc9-1* strain may provide evidence for a complementary role for Rts1 function in allowing replication fork progression. In *cdc9-1* strains, increased DNA damage is seen due to the collapse of replication forks. This results in double strand breaks (DSBs), and the DNA damage response (DDR) being activated. One of the ways to repair DSBs is via break-induced replication (BIR), which is activated in *cdc9-1* cells ( Vasianovich *et al.*, 2014). A role for Rts1 in recruiting PP2A phosphatase to control phosphorylation steps in the DDR, potentially in BIR, could be hypothesised. In this instance, *rts1Δ* in a *cdc9-1* background would give increased lethality, since there would also be an impaired capacity for cells to repair *cdc9-1* dependent DSBs.

Evidence in support of the synthetic lethality of *RTS1* and DNA replication factors can be found on BioGRID, a summary of published genetic interactions in budding yeast. A combination of high and low throughput genetic interaction screens show that *RTS1* exhibits negative genetic interactions (a term reserved for genetic screens which show a more lethal phenotype in strains where two mutations are combined than in the respective single mutant strains) with *DBF4*, *CDC6* and *ORC6* and *DPB11* ( Archambault *et al.*, 2005; Collins *et al.*, 2007; Costanzo *et al.*, 2016). However, we show here, for the first time, negative genetic interactions of *RTS1* with *CDC45* and *CDC9*.

## Conclusions

Given the wealth of recent literature outlining the importance of Rif1 in opposing the actions of DDK kinase, it is clear that phosphatases play an important role in controlling DNA replication origin firing. However, we do not find evidence for an analogous role for PP2A, specifically via its regulatory subunit Rts1. Deletion of *RTS1* in combination with mutations in origin licensing factor genes, *ORC* and *CDC6*, showed little or no genetic interaction, providing no genetic evidence for Rts1-PP2A controlling DNA replication origin licensing. Further, we found no role for Rts1 in opposing DDK phosphorylation. However, we observed some level of increased temperature-sensitivity phenotype when *RTS1* was deleted in many of the replication initiation factor mutant strains, alluding to a potential synthetic lethality phenotype. Increased temperature-sensitivity was most pronounced in late-acting DNA replication firing factors ** Cdc45 and Dpb11. Additionally, an increased requirement for *RTS1* in a *cdc9-1* background was found. We speculate that a functional overlap between Rts1-PP2A and Cdc9 may exist via replication fork progression mechanisms. Rts1 may recruit PP2A during BIR, or the DDR, in response to DSBs. Over-expression of *RTS1* could potentially compensate for the increased replication fork collapse seen in *cdc9-1* mutants, giving synthetic rescue of temperature-sensitivity. Further studies would be needed to confirm this hypothesis.

## Data availability

*All data underlying the results are available as part of the article and no additional source data are required*.
