# Supplementary material for: Investigating the role of Rts1 in DNA replication initiation
Source: Wellcome Open Res. 2018 Mar 6;3:23. [Version 1] doi: 10.12688/wellcomeopenres.13884.1 (PMC5897792; doi:10.12688/wellcomeopenres.13884.1)
Supplement: Peer review history

# Peer review: Referee response for version 1

**DOI:** 10.21956/wellcomeopenres.15094.r31563

**Published:** 2018-04-10

**Authors:** David Shore, Maksym Shyian

**Affiliations:** 1 Department of Molecular Biology & Institute of Genetics and Genomics in Geneva (iGE3), University of Geneva, Geneva, Switzerland

Briefly, this study presents a single negative result (absence of *orc2*, *cdc7*, of *dbf4* rescue by *rts1* *Δ*) already available for the scientific community in Costanzo et al., 2016. The authors should provide additional information or perform experiments to confirm reproducibility of the data, especially of the only novel finding they report.

 Comments:

1. Page 3, lanes 15-16 of the Introduction: ‘…and the replicative polymerases (Polε, Polδ and Polα) (Yeeles et al., 2015)’ is a speculation and should be corrected, as Cdc45, Sld2, Sld3, Dpb11 are not known to recruit polymerases alpha and delta. Moreover, the biochemical study Yeeles et al., 2015 used as a reference did not include polymerase delta in the reaction at all.
2. Materials and Methods: transformation of yeast is mutagenic and subsequent selection of transformants may lead to the isolation of unnecessary background mutations affecting the growth of the strain. Therefore, it is advisable to do transformations in diploid strains with subsequent isolation of haploid combinations by sporulation and tetrad dissection ^1^. Thus, the authors should clarify whether the *rts1* *Δ* and *rif1* *Δ* transformations were done in haploid or diploid strains. Were haploid deletions obtained by sporulation of heterozygous diploids obtained by transformation? If so, how many different spore colonies were assessed for the ts phenotypes in each case to make sure that the growth phenotypes are reproducible and consistent? (Diploid mutant strains are indicated in the strain list, but their genotypes are not correctly described so as to indicate whether they are heterozygous or homozygous mutants. Correct nomenclature should be used.)
3. Discussion, page 9, lane 7: ‘HSP60’ and ‘Hsp60’ should be written instead of ‘HPS60’ and ‘Hps60’
4. Curiously, the Costanzo et al., 2016 study and BioGRID do not detect/contain data on negative genetic interactions of *rts1* *Δ* (nor *pph21* *Δ/22* *Δ*) with *cdc45-ts*. There are slight negative interactions for *rts1* *Δ* with either *cdc9-1* or *dpb11-1*, but these were also reported to contain suppressor mutations that might complicate the picture (see TheCellMap web site). Could it be because the above studies used other alleles of these genes? The authors may try to compare various ts alleles of *CDC45*, *DBP11*, and *CDC9* to see if there are allele-specific interactions of *rts1* *Δ*. Since the authors claim in the Discussion section that this is the main novelty in their study, the use of other ts alleles, degron-based alleles or the TET-off system is advised to make this point stronger.
5. As Cdc9 participates in lagging strand DNA synthesis while Dpb11 works at the leading strand, other mutants affecting leading and lagging strand synthesis should be checked in order to clarify the matter and try and place RTS1 in one or both of these DNA synthesis pathways.
6. The proposition to overexpress *RTS1* in *cdc9-1* should be directly carried out by the authors of this study.
7. Is *SGO1* involved in the *RTS1* genetic interaction with *CDC45*/ *DBP11*/ *CDC9*? How will *sgo1* *Δ* affect the ts alleles of these genes?

We have read this submission. We believe that we have an appropriate level of expertise to confirm that it is of an acceptable scientific standard.

## References

[1] DunhamMJGartenbergMRBrownGW: Methods in yeast genetics and genomics, 2015 edition: a CSHL course manual.2015;

# Peer review: Referee response for version 1

**DOI:** 10.21956/wellcomeopenres.15094.r31562

**Published:** 2018-03-22

**Authors:** Gilles Fischer

**Affiliations:** 1 French National Centre for Scientific Research (CNRS), Institute of Biology Paris-Seine, Laboratory of Computational and Quantitative Biology (LCQB) UMR7238, Pierre and Marie Curie University (UPMC)-Sorbonne University, Paris, France

The hypothesis that underlies this work is that *RTS1,* the regulatory unit of the PP2A phosphatase, could counteract the initiation of DNA replication through dephosphorylating initiation factors, in a way similar to the action of *RIF1-*PP1 on MCM. However, this hypothesis is very fragile as it solely relies on a positive interaction between *rts1* *-delta* with a *ts-orc2* mutant, which was previously reported in a large-scale genomic screen for genetic interactions (Constanzo et al., 2010). Firstly, the score actually reported in the CellMap website (thecellmap.org) for this positive interaction is very low (0.07), below the default confidence threshold set for this study (0.08). Secondly, such large-scale genomic screens are often populated with a large number of weakly supported false positives. Therefore, it is not very surprising that a careful examination of an originally weakly supported interaction could lead to a different result. Thirdly, the authors also mention that more a recent screen reported opposite observations (enhanced phenotype in some replication mutants). Finally, the authors also indicate in the introduction that Rts1 is required for Cdc45 loading onto chromatin, which is not very compatible with their initial hypothesis that Rts1 could counteract replication initiation. Altogether these elements show that the initial hypothesis onto which the manuscript is constructed is not really reliable. I would suggest reformulating the manuscript and presenting equitably to two alternative hypotheses in the introduction, namely Rts1 is promoting or opposing replication initiation. I would also clearly state that Rif1 is used as a control to discriminate between the 2 hypotheses because as it is now presented, the reason why a parallel is made between the role of Rif1 and that of Rts1 is not really evident.

 I have few other minor concerns. I found the title to be a bit misleading because this work does not directly assess the role of *RTS1* in DNA replication initiation per se but rather at the genetic interactions between this gene and other initiation factors.

 I had a hard time trying to understand the logic of Fig 1. I think the legend could be more explicit to guide the reader through the different cases.

 I would also suggest to replace throughout the text 'synthetic lethality' by 'negative interaction' because synthetic lethality is generally considered as an extreme case of a negative interaction where two mutations, each causing limited fitness defect on their own, result in a inviable phenotype. I would also replace 'synthetic rescue' by 'positive interaction' for the same reason. This would allow avoiding turn of phrases such as 'greater synthetic lethality'.

 In the first paragraph of the result section, the authors mention that an orc2-1 mutant was reported to show growth deficiency even at permissive temperature. However, the spotting assay in Fig. 2A does not show any growth defect at restrictive temperature for the single orc2-1 mutant.

 In its entirety, this study confirms a number of phenotypes previously described, including the opposing actions of *RIF1 *and DDK on replication initiation, which confirms that the experiments are carefully performed. It also highlights some new observations such as a small positive interaction between *orc2-1* and *rif1. *The controls are adequate (comparison of WT and all single and double mutants) and the described data are convincing. The introduction section is really well written and give a very good overview of the interplay between the various actors involved in replication initiation. All in all, the study is a valuable additional contribution to the field of DNA replication.

I have read this submission. I believe that I have an appropriate level of expertise to confirm that it is of an acceptable scientific standard.
